# Supplementary material for: Distribution and Activity of Sulfur-Metabolizing Bacteria along the Temperature Gradient in Phototrophic Mats of the Chilean Hot Spring Porcelana
Source: Microorganisms. 2023 Jul 14;11(7):1803. doi: 10.3390/microorganisms11071803 (PMC10385741; doi:10.3390/microorganisms11071803)
Supplement: Supplementary file 1 [file microorganisms-11-01803-s001.zip › Supplementary_figures.pdf]

Distribution and activity of sulfur-metabolizing bacteria along the temperature gradient of Porcelana phototrophic hot spring mats

Ricardo Konrad[1], Pablo Vergara-Barros[1], Jaime Alcorta[1], María E. Alcamán-Arias[2,3,4], Gloria Levicán[5], Beatriz Díez[1,3,6*]

[1]*Department of Molecular Genetics and Microbiology, Biological Sciences Faculty, Pontifical Catholic University of Chile, Santiago, Chile.*

[2]*Department of Oceanography, University of Concepcion, Concepcion, Chile.*

[3]*Center for Climate and Resilience Research (CR)2, Chile*

[4]*Escuela de Medicina, Universidad Espíritu Santo, Guayaquil 0901952, Ecuador*

[5] *Biology Department, Chemistry and Biology Faculty, University of Santiago of Chile, Santiago, Chile*

[6]*Center for Genome Regulation (CGR), Chile*

Corresponding author email/address:

bdiez@bio.puc.cl

Department of Molecular Genetics and Microbiology, Pontifical Catholic University of Chile, Avenida Libertador Bernardo O'Higgins 340, Casilla 144-D, C.P. 651 3677, Santiago, Chile.

Keywords: sulfur oxidation-reduction, soxB, aprA, 16S rRNA, phototrophic hot spring mat, metagenomics

Supplementary Table Legends

Table S1: PCR primer sequences for aprA and soxB and PCR parameters., Table S2: porcelana hot spring physicochemical parameters.

Table S3: gene names and KO IDs of sulfur cycle-associated pathways.

Table S4: gene counts for sul-fur-associated KO IDs grouped at order level in metagenomes and metatranscriptomes obtained from Porcelana hot spring.

Table S5: aprA and soxB CDS recovered from metagenomes and OTUs from Porcelana hot spring.

Table S6: Genomic taxonomy of aprA- or soxB-containing MAGs ob-tained from Porcelana hot spring according GTDB r89.

a) *apr*A

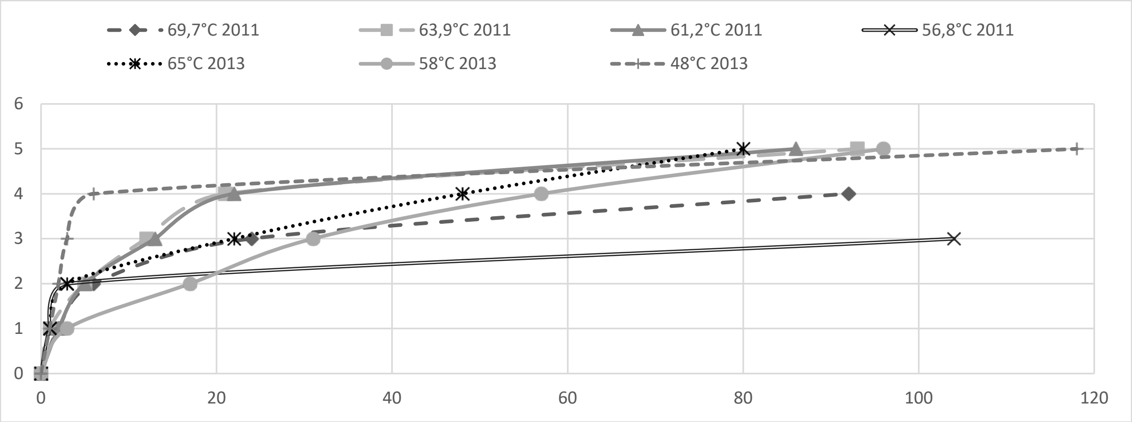

b*)* *sox*B

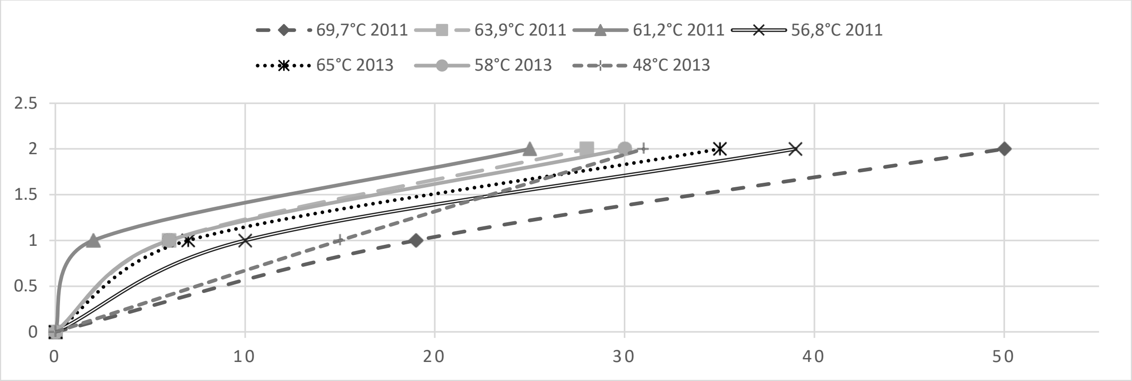

Figure S1. Rarefaction curves for *apr*A (a) and *sox*B (b) genes.
